# Fatty-Acid Binding Proteins Modulate Sleep and Enhance Long-Term Memory Consolidation in *Drosophila*

**DOI:** 10.1371/journal.pone.0015890

**Published:** 2011-01-27

**Authors:** Jason R. Gerstner, William M. Vanderheyden, Paul J. Shaw, Charles F. Landry, Jerry C. P. Yin

**Affiliations:** 1 Department of Genetics, University of Wisconsin-Madison, Madison, Wisconsin, United States of America; 2 Anatomy and Neurobiology, Washington University School of Medicine, St. Louis, Missouri, United States of America; 3 Scarab Genomics, LLC, Madison, Wisconsin, United States of America; 4 Department of Neurology, University of Wisconsin-Madison, Madison, Wisconsin, United States of America; 5 Waisman Center, University of Wisconsin-Madison, Madison, Wisconsin, United States of America; Université de Bordeaux and Centre National de la Recherche Scientifique, France

## Abstract

Sleep is thought to be important for memory consolidation, since sleep deprivation has been shown to interfere with memory processing. However, the effects of augmenting sleep on memory formation are not well known, and testing the role of sleep in memory enhancement has been limited to pharmacological and behavioral approaches. Here we test the effect of overexpressing the brain-type fatty acid binding protein (Fabp7) on sleep and long-term memory (LTM) formation in *Drosophila melanogaster*. Transgenic flies carrying the murine Fabp7 or the *Drosophila* homologue dFabp had reduced baseline sleep but normal LTM, while Fabp induction produced increases in both net sleep and LTM. We also define a post-training consolidation “window” that is sufficient for the observed Fabp-mediated memory enhancement. Since Fabp overexpression increases consolidated daytime sleep bouts, these data support a role for longer naps in improving memory and provide a novel role for lipid-binding proteins in regulating memory consolidation concurrently with changes in behavioral state.

## Introduction

Although sleep is an essential behavioral process conserved across phyla from fruit flies to humans, its functions remain elusive. There are many theories about the function of sleep, including roles in metabolic balance [1], excitotoxic repair [2], and memory consolidation [3]. The process of memory is also widely conserved in the animal kingdom, and the relationship between sleep and memory formation continues to be controversial [4].

Studies in both rodents [5] and Aplysia [6] show a clear time-of-day effect on memory formation. In flies, short-term memory has been shown to be regulated by processes involving both circadian rhythms [7] and sleep [8]. In addition, a temporal window for the effects of sleep deprivation on memory has been shown in both invertebrate and vertebrate species. Four hours of sleep deprivation (SD) immediately following courtship training abolishes memory retention in flies [9], while SD later during the sleeping period has no effect. In rodents, REM SD either just before [10], or immediately after behavioral training [11], has been shown to abolish contextual (hippocampal dependent) memory formation. It has also been suggested that REM sleep is necessary during later consolidation periods of memory in rodents [12]. While these results imply a role for sleep in the consolidation of memory, they describe the consequences of reducing sleep (or specific components of sleep) on the formation of memory. An alternate method for understanding the influence of sleep on memory formation would be to test the effects of augmenting sleep using genetic approaches. While molecular targets involving circadian- and sleep-dependent memory formation are beginning to be identified [13], [14], specific molecular targets responsible for augmenting sleep and memory concomitantly are not known.

A number of laboratories have used microarrays to identify sleep (or deprivation)-responsive genes [15]–[19], and some of these include immediate-early genes (IEGs), many of which have been shown to be involved in learning and memory consolidation [20]–[22]. Levels of some IEGs, such as Zif268, are elevated in response to enriched environments or long-term potentiation (LTP) during subsequent REM sleep episodes in rodents [23], [24]. The regulation of synaptic plasticity-related molecules in sleep/wake behavior suggests that the sleep/wake system may re-deploy some of the molecular components underlying learning and memory [25]. Previously we used microarray analysis to show that brain-type fatty-acid binding protein (Fabp7) shows circadian oscillation in its expression [26], and that its cycling pattern is widespread throughout mammalian brain [27]. Here we utilize the *Drosophila* model to investigate the functional role of fatty-acid binding proteins in sleep and memory consolidation, and describe a novel molecular player for improving memory through increases in daytime sleep.

## Results

### Characterization of *Drosophila* Fabp

**Figure 1:**
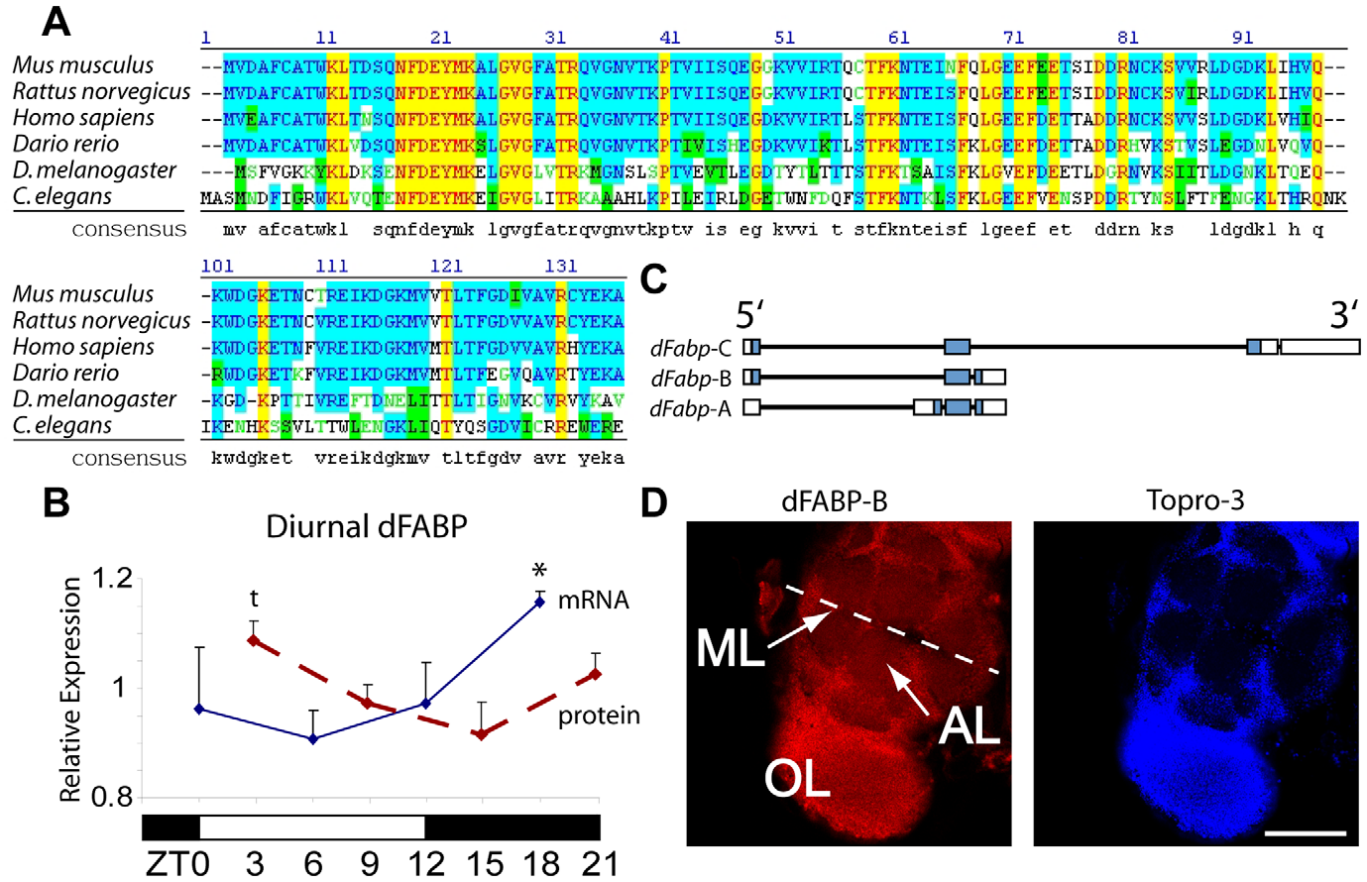
Characterization and diurnal regulation of the *Drosophila* fatty-acid binding protein, dFabp. (A) Amino acid sequence homology of dFabp is compared to brain-type fatty-acid binding protein (Fabp7) of mouse, rat, human, zebra fish, and worm (lbp-7). A consensus sequence of all species is also shown. (B) Densitometric analysis of Northern and Western blots reveals a diurnal regulation of dFabp mRNA (blue solid line) and protein (red dashed line) in fly head. dFabp mRNA levels are normalized to Gapdh, with elevated expression during the sleep period (mean ± s.e.m., N = 2/timepoint, 50–100 heads per timepoint, asterisk *P*<0.05, *t*-test ZT18 vs ZT6). dFabp protein levels are normalized to laminin, and are elevated toward wake onset (mean ± s.e.m., N = 4/timepoint, 50–100 heads per timepoint, t *P* = 0.051, *t*-test ZT3 vs ZT15). Open bar  =  lights on, filled bar  =  lights off. (C) Schematic representation of the dFabp locus. Open bar  =  non-coding, filled bar  =  coding region. (D) Projection slice of brain stained with an antibody against dFabp (red), and the nuclear stain Topro-3 (blue), as viewed by confocal microscopy. ML  =  midline, OL  =  optic lobe, AL  =  antennal lobe, scale bar  = 120 mm.

Fatty-acid binding proteins (Fabps) comprise a group of soluble proteins that bind small hydrophobic lipids and act as transporters. The mammalian Fabp family consists of ∼9 separate genes, which are expressed in various cell and tissue types. In the adult mammalian central nervous system (CNS), only three of these appear to be expressed, and include heart-, epidermal-, and brain-type fatty-acid binding proteins (Fabp3, 5, 7, respectively). In contrast, there is a single Fabp locus (CG6783) in the *Drosophila* genome, which we have termed dFabp, that encodes at least 3 alternatively spliced isoforms of *Drosophila* Fabp (dFabp) (Fig. 1C). The second dFabp isoform (CG6783-B) shares the greatest homology to mouse Fabp7 (54% Identical, 68% Positive). An alignment of the amino-acid sequences for Fabps from various species, and a derived consensus sequence, are shown in Fig. 1A.

Since the mouse Fabp7 transcript and protein have been shown to follow diurnal changes in expression, we asked whether dFabp also oscillates across the day/night cycle. Analysis of dFabp mRNA from fly head extracts show changes based on time-of-day, which reflect circadian changes previously observed [28]. We identified an elevation in mRNA levels during sleep and reduced levels during the wake period (Fig. 1B). The increase in dFabp mRNA preceded an accumulation of dFabp protein, which peaked early in the waking phase (Fig. 1B; Figure S1A). These results mirror oscillations in Fabp7 expression observed in mouse brain [27], suggesting a conserved function over the day/night cycle. Immunocytochemical (ICC) analysis using antibodies raised against dFabp-B revealed a broad expression pattern throughout the fly brain (Fig. 1D). ICC staining against dFabp appeared to be much stronger in nuclei (identified using the nuclear marker Topro-3) than in the cytoplasm (Fig. 1D), however the signal cannot be verified to be solely due to endogenous expression.

### Fabp expression regulates sleep and memory

**Figure 2:**
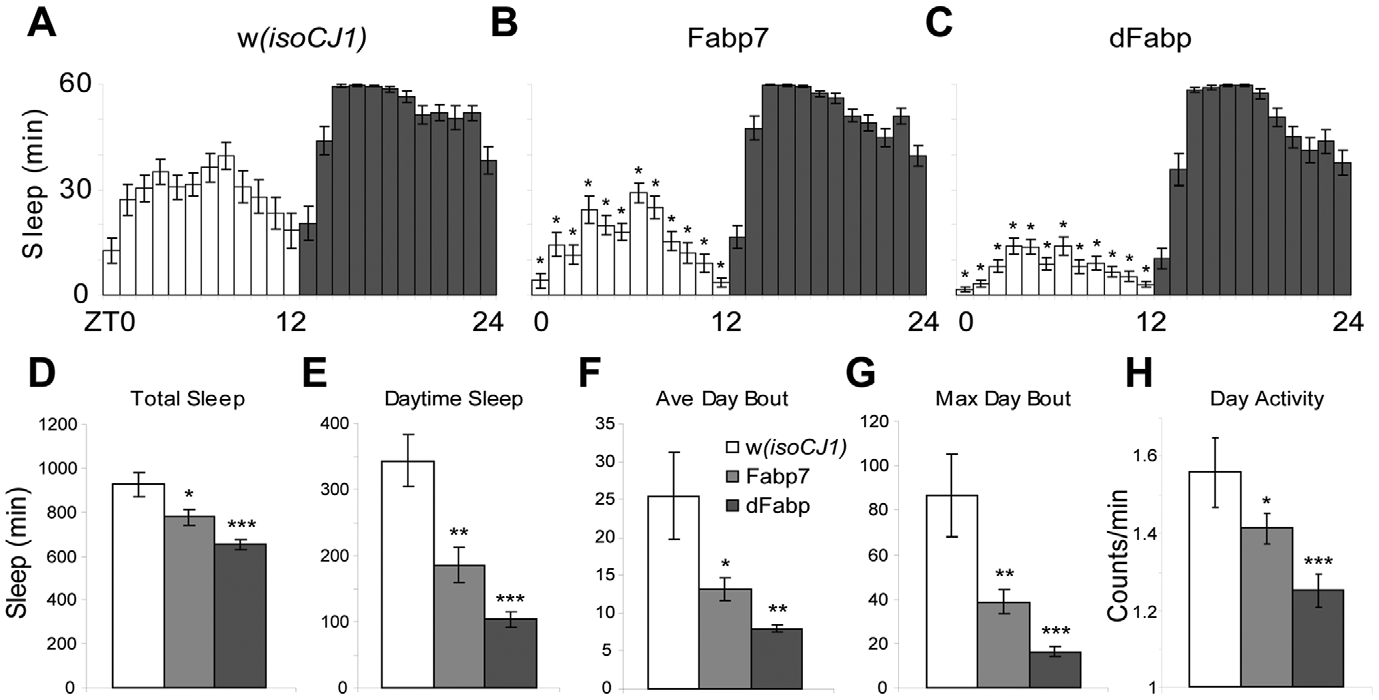
Reduced sleep in Fabp7 and dFabp overexpressing flies. Hourly diurnal sleep profile of the (A) *w(isoCJ1)* background strain, (B) Fabp7, and (C) dFabp flies. Open bars  =  lights on, filled bars  =  lights off. Analysis of various sleep parameters: (D) Total sleep over 24 hours, (E) Total daytime sleep, (F) Average daytime sleep bout length, (G) Maximum daytime sleep bout length, (H) Daytime locomotor activity per waking minute. Open bars  =  *w(isoCJ1)*, light grey bars  =  Fabp7, dark grey bars  =  dFabp. Results are mean ± s.e.m.; *n* = 23–24, representative of 3–5 experiments. Single asterisk, *P*<0.05; double asterisk, *P*<0.01; triple asterisk, *P*<0.001, *t*-test.

Genes that are involved in regulating behavioral states (wake, sleep) might be predicted to show coordinated, brain-wide changes in their expression levels across the day/night cycle. Since both flies (Fig. 1B) and mammals [27] exhibit a diurnal fluctuation of lipid-binding protein expression in the CNS, we examined the effects of altering Fabp expression on sleep behavior. We generated heat-shock inducible transgenic flies carrying the mouse Fabp7 open reading frame (ORF) in the background strain *w(isoCJ1)*, an isogenized wild-type strain. Western blots probed with species-specific antibodies showed that the transgenic flies overexpress protein, even at lower temperatures (20°C; Figure S1B). Surprisingly, this overexpression of Fabp7 resulted in a dramatic reduction in baseline sleep relative to the *w(isoCJ1)* background flies (housed at ∼22°C), principally from decreased daytime sleep (Fig. 2B, D, E). These effects on baseline sleep also occur in male flies (data not shown) and two additional lines carrying the Fabp7 ORF (Figure S2). We observed similar reductions in total and daytime sleep in transgenic flies that carry the *Drosophila* dFabp-B ORF (Fig. 2C, D, E). The average daytime bout length and the average maximum daytime bout length are both significantly reduced in Fabp7 and dFabp flies (Fig. 2E, F). The bout length is considered to be a measurement of consolidated sleep. Two additional dFabp overexpressing fly lines also showed similar significant results in each of these sleep parameters (Figure S3). The reduction in daytime sleep was not due to hyperactivity, since daytime locomotor activity, as measured by counts per waking minute, was in fact reduced in Fabp7 and dFabp flies (Fig. 2G; Figures S2G; S3G). The total number of sleep bouts over a 24-hour period for the transgenic flies was not different from controls (data not shown).

**Figure 3:**
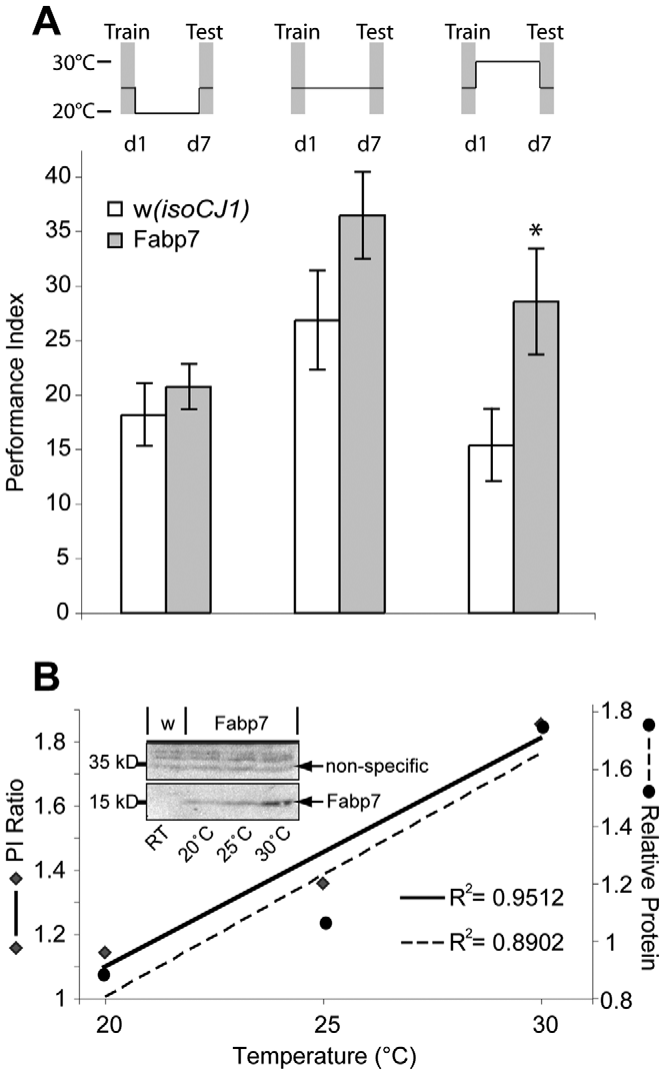
Fabp7 overexpression enhances 7-day long-term memory (LTM). (A) Post-training induction of the heat shock (HS) promoter-driven Fabp7 flies produces a temperature-dependent enhancement of memory after 10x spaced training if flies are maintainted at higher temperatures (30°C) for 7 days prior to testing. Grey bars, Fabp7, open bars, *w(isoCJ1)* background strain. Results are mean ± s.e.m.; *n* = 8–10 groups. Asterisk *P*<0.05, *t*-test. (B) Ratio of Performance Index (PI) scores (A) for Fabp7/*w(isoCJ1)* (diamonds; *y*-axis left) shows a correlation of increasing performance with temperature (R^2^ = 0.95), similar to the correlation of increase in protein expression (black circles; *y*-axis right) with temperature (R^2^ = 0.89). Inset: Western blot of Fabp7 protein expression in Fabp7 staged at 20°, 25°, and 30°C for 7 days, and the immunoreactive background of *w(isoCJ1) (w)* at room temperature (RT), in nuclear fraction (lysate sample: ∼50–100 fly heads per lane).

Given the reduction in daytime sleep in Fabp7 and dFabp flies, and the growing appreciation for the importance of sleep in memory formation, we tested the effect of altering post-training Fabp protein levels on long-term memory formation. Wild type and transgenic flies were trained with 10 cycles of spaced training, and then stored at various temperatures to modulate the post-acquisition levels of Fabp protein expression. Despite the reduced baseline sleep phenotype observed in Fabp flies at lower temperatures (Fig. 2), trained flies maintained at 20°C did not show any changes in performance as compared to the *w(isoCJ1)* background strain, (Fig. 3A). However, inducing Fabp7 overexpression in flies stored at 30°C during the maintenance period following training showed a significant enhancement of 7-day LTM (Fig. 3A). There was a strong correlation between temperature and memory when the ratio of performance between Fabp7: *w(isoCJ1)* flies was plotted, and this paralleled the increase in Fabp7 protein expression (Fig. 3B). Learning scores for Fabp7 flies were comparable to the background strain *w(isoCJ1)* (Figure S4A), further supporting the conclusion that the temperature-induced enhancement of memory was not due to an effect on learning. This result suggests that some aspect of Fabp overexpression during the 7-days following training enhances memory formation.

**Figure 4:**
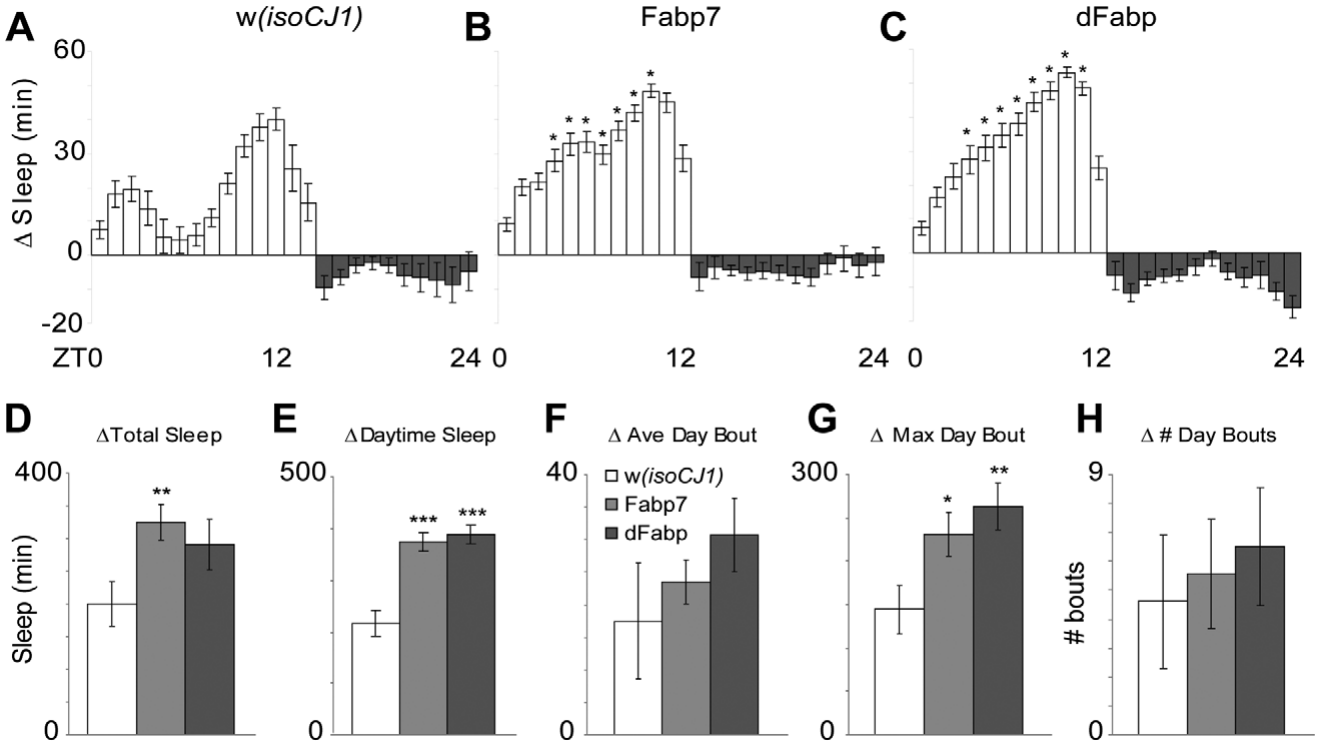
Increased net sleep in Fabp7 and dFabp overexpressing flies. Hourly change in sleep profile of the (A) *w(isoCJ1)* background strain, (B) Fabp7, and (C) dFabp flies shifted from 20°–30°C. Open bars  =  lights on, filled bars  =  lights off. Analysis of change in various sleep parameters of flies shifted from 20°–30°C: (D) Total sleep over 24 hours, (E) Total daytime sleep, (F) Average daytime sleep bout length, (G) Maximum daytime sleep bout length, (H) Number of daytime bouts. Open bars  =  *w(isoCJ1)*, light grey bars  =  Fabp7, dark grey bars  =  dFabp. Results are mean ± s.e.m.; *n* = 20–30. Single asterisk, *P*<0.05; double asterisk, *P*<0.01; triple asterisk, *P*<0.001, *t*-test.

Since the transgenic flies showed a temperature-dependent enhancement of memory formation, we examined whether there are changes in sleep as a function of temperature. A significant increase in total sleep occurred in Fabp7 flies when the transgene was induced, compared to *w(isoCJ1)* flies (Fig. 4B, D). This increase in total sleep resulted primarily from increases in daytime sleep (Fig. 4E), since night-time sleep was similarly reduced in all flies (Fig. 4A, B, C). A more detailed analysis showed that Fabp7 and dFabp transgene induction increased the maximum daytime bout length (Fig. 4G), without significantly affecting the average daytime bout length (Fig. 4F) or bout number (Fig. 4H), compared to *w(isoCJ1)* controls. Therefore, increasing the temperature from 20°C to 30°C increased Fabp protein expression and correlated with a net increase in longer daytime sleep bouts.

### Temporal window for Fabp-mediated memory enhancement

In order to further determine when, after behavioral training, Fabp7 and dFabp act to enhance memory formation, we tested various temporal “windows” of overexpression. It has previously been shown that aPKM transgenic flies enhance memory formation when the transgene is induced shortly after the end of behavioral training [29]. However, increasing Fabp7 immediately after the end of LTM training did not enhance 24-hour memory (data not shown).

**Figure 5:**
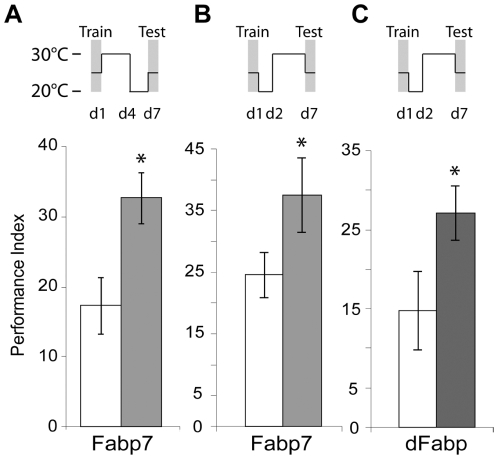
Fabp7- and dFabp-mediated LTM enhancement is preserved in a late consolidation period. (A) Fabp7 (grey fill) flies show a significant enhancement in 7 day LTM performance after 10X Spaced training, when shifted from 30°–20°C on day 4, compared to *w(isoCJ1)* background flies (open fill bar). Results are mean ± s.e.m.; *n* = 6 groups. Asterisk *P*<0.05, *t*-test. (B) Fabp7 (light grey fill) and (C) dFabp (dark grey fill) flies show a significant enhancement in 7 day LTM performance after 10X Spaced training, when shifted from 20°–30°C on day 2, as compared to *w(isoCJ1)* background flies (open fill bars, B,C). Results are mean ± s.e.m.; *n* = 7–8 groups. Asterisk *P*<0.05, *t*-test. d1  =  day 1, d2  =  day 2, d4  =  day 4, d7  =  day 7.

Next we tested if Fabp7 overexpression during the first four days after training is sufficient to enhance seven day LTM formation. Flies were trained at 25°C and immediately placed at 30°C for four days, after which they were shifted back to 20°C for the remaining time. This incubation schedule preserved memory enhancement (Fig. 5A), suggesting that the effects of Fabp7 on LTM require sustained periods of overexpression. Shifting the overexpression window to a later consolidation period (between post-training days 2 and 7) also preserved enhancement of LTM in Fabp7 flies (Fig. 5B). Further, this effect replicated when we analyzed dFabp flies using this same regimen (Fig. 5C). While Fabp flies showed elevated olfactory avoidance responses following five days at 30°C (data not shown), we still observed an increase in LTM when Fabp7 flies were shifted back to 20°C for two days prior to testing (Fig. 5A). This result suggested that elevated Fabp expression for the time period just prior to recall (Fig. 5B, C) is not responsible for the increase in LTM observed. However, in order to rule out the possible effects of Fabp on sensory-systems during retrieval, we examined the effects of increasing Fabp7 on memory recall. We found that an acute induction of Fabp7 two hours prior to testing for LTM actually inhibited performance (Figure S4C). This suggests that acute Fabp expression at the time of retrieval is able to impede memory recall processes. Together, these data provide evidence that the Fapb7-mediated enhancement effects on LTM occur during the consolidation period, and are not due to effects on learning, acquisition or recall.

**Figure 6:**
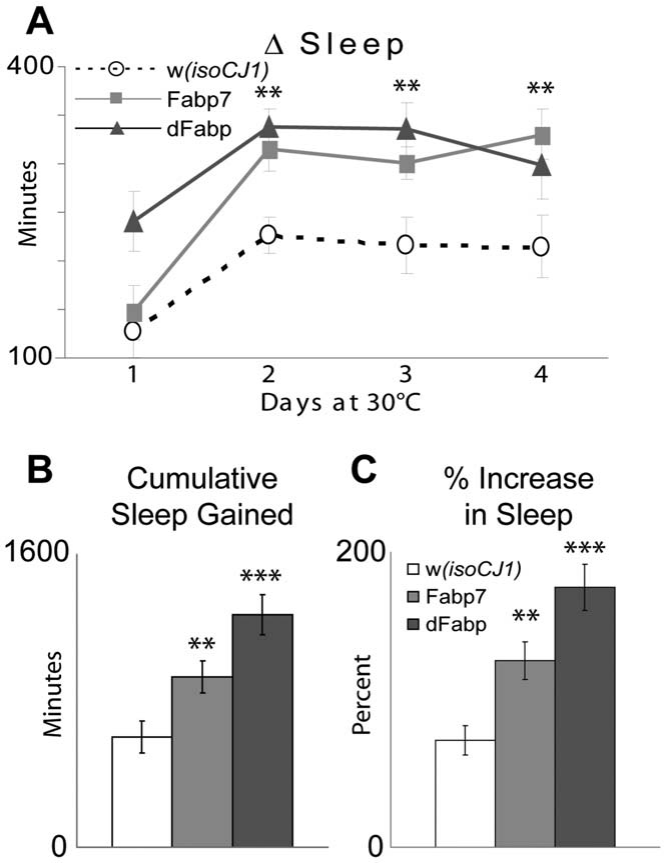
Cumulative increased sleep in Fabp7 and dFabp flies correlates with LTM enhancement. (A) Fabp7 (light grey square, solid line) and dFabp (dark grey triangle, solid line) flies have an increase in sleep above the *w(isoCJ1)* background strain (open circle, dashed line) following a shift from 20°–30°C. Results are mean ± s.e.m.; *n* = 20–30; double asterisk *P*<0.01, (Fabp7 vs. *w(isoCJ1)*), *t*-test. (B) Cumulative increases in minutes of total sleep gained and (C) percentage of sleep increased over four days when maintained at 30°C are higher in Fabp7 and dFabp flies, as compared to the *w(isoCJ1)* background strain. Open bars  =  *w(isoCJ1)*, light grey bars  =  Fabp7, dark grey bars  =  dFabp. Results are mean ± s.e.m.; *n* = 20–30; double asterisk *P*<0.01, triple asterisk *P*<0.001, *t*-test.

We have shown that at low temperatures where the transgene induction is mild, the Fabp7 or dFabp proteins reduce the steady-state amount of daytime sleep. However, when the transgenes are induced to higher levels, the net gain in daytime sleep that flies obtained (relative to their isogenic control line) was substantial. Memory formation was enhanced when flies were stored at inducing temperatures. Is the total amount of sleep gained correlated with the memory enhancement? To test if these two phenotypes are correlated, we measured the cumulative change in sleep of Fabp7 and dFabp flies maintained at 30°C for four consecutive days following two days baseline at 20°C. This is the temperature regimen that produces enhancement of memory formation during the consolidation phase. There was a significant increase in minutes and percentage of sleep gained in Fabp7 and dFabp flies, as compared to *w(isoCJ1)* flies, on the second, third, and fourth days at 30°C (Fig. 6A). In addition, the cumulative increase in total sleep (Fig. 6B), and percentage of total sleep gained (Fig. 6C) was significantly higher in both Fabp7 and dFabp flies compared to *w(isoCJ1)* flies for the four days at 30°C. Taken together, these results show that at elevated temperatures following spaced training, Fabp7 and dFabp flies undergo an increase in cumulative sleep during the consolidation period, and this increase correlates with an enhancement in LTM performance.

## Discussion

Previously we have shown that the mRNA levels for Fabp7 are diurnally regulated and are highest during the sleeping period of mice [26], [27]. Here we characterized the Fabp7 *Drosophila* homologue dFabp, and show that dFabp mRNA expression also cycles across the day, with elevated levels during the sleeping period. Further, in order to determine whether increasing Fabp expression influences complex behavior, we show that induction of Fabp7 or dFabp in transgenic flies is sufficient to produce sleep and long-term memory enhancement phenotypes. These data suggest a common theme for cycling molecules in the formation of long-term memory, and suggest that like other known circadian genes, Fabp-mediated signaling is likely implicated in both sleep/wake and memory-related processes.

### Regulation of Fabp expression

What are the upstream molecular signaling cascades currently known to regulate Fabp7 expression? While the characterization of specific molecular pathways involved in modulating Fabp7 levels across the day/night cycle has not yet been reported, *cis*-acting elements regulating Fabp7 expression during CNS development have begun to be identified. Pax6, a transcription factor known to promote developmental neuronal differentiation, was shown to regulate Fabp7 expression, in a manner believed to be critical for the proper maintenance of neuroepithelial cells [30]. Fabp7 has also been shown to be a downstream target of Notch signaling pathways during CNS development [31]. Since Notch signaling has also been implicated in LTM formation in both rodents [32] and flies [33], [34], the Notch cascade could represent a conserved molecular pathway mediating Fabp-induced memory-related processes in the adult CNS across animal phylogeny. Since Notch-effector binding sites are observed in both mammalian and fly promoters for Fabp7 [31] and dFabp (J. Gerstner, unpublished observations), respectively, future studies aimed at the identification of these and other phylogenetically conserved *cis*-acting elements will provide novel targets for sleep and cognitive therapeutics.

Fabp7 knock-out (KO) mice have previously been shown to have altered behavioral responses, including an increase in anxiety-like behavior [35], and deficits in prepulse inhibition, a hallmark of schizophrenia [36]. Fabp7 mRNA expression is known to be enriched in the dendritic layers of the hippocampus [37], where it has also been shown to be induced following kainate injection [38], a procedure that initiates epileptoform activity. Further, following the induction of a constitutively active form of CREB, Fabp7 mRNA shows an elevation in expression within the hippocampus that mirrors the temporal profile of CREB-induced BDNF mRNA expression [39]. Taken together, these data suggest a role for Fabp7 in plasticity-related mechanisms in mammals, and implicate Fabp7 in pathways downstream of well established memory-related molecular signaling cascades.

### Genes, sleep and memory

There is an emerging notion that sleep, like the circadian system, is under genetic control [40], [41]. In addition, mounting evidence suggests that genes involved in the circadian system are able to influence memory processes [42], [43]. For example, disruption of core clock components (such as CLOCK and BMAL1) has been shown to inhibit long-term memory in mice [44]. In *Drosophila*, a null mutation of the circadian gene *period* blocked memory, while overexpression of the *period* gene protein product PER was able to enhance memory formation [45]. Recently it has been shown that *period* gene expression was also able to regulate experience-dependent increases in sleep in *Drosophila* [13]. These data are interesting because they suggest that transcription of specific genes that are known regulators of circadian rhythms are able to regulate other complex neurological phenomena, such as learning and memory formation.

Interestingly, while Fabp7 KO mice have not yet been examined for changes in circadian rhythms or behavioral state, KO mice for another Fabp expressed in the mammalian brain, Fabp3, have recently been shown to have increased locomotor activity over the light-dark cycle [46]. Further, Fabp3 has been shown to interact with the dopamine receptor (D2R), and is highly expressed in acetylcholine interneurons and at glutamatergic terminals [46], implicating lipid-transport pathways in the regulation of dopaminergic signaling within the CNS. Together, these data suggest that lipid-mediated pathways could modulate diurnal changes in locomotor activity, perhaps through a Fabp-regulated mechanism involving signaling cascades downstream of D2R activation.

### Influence of lipid metabolites on sleep and memory

Prostaglandin (PG) D2 is lipid-derived eicosanoid that has been shown to promote sleep following the activation of PGD2 receptors (DP1R) and subsequent release of adenosine [47]. In rats, cerebrospinal fluid levels of PGD2 have been shown to exhibit a circadian rhythm [48], and also increase following sleep deprivation [49], suggesting that PGD2 regulates normal sleep in mammals. Infusion of PGD2 into the third ventricle of rats during their wake-period resulted in a dose-dependent increase in both REM and NREM sleep [50]. Dose-dependent increases in sleep were also observed when PGD2 was infused into the lateral ventricle of wild-type (WT) mice, but not in DP1R KO mice [51]. Pharmacological inhibitors of the enzyme which produces PGD2 out of arachidonic acid derivatives, PGD synthase (PGDS), generate a reversible and dose-dependent inhibition of sleep in rats [52]. Interestingly, PGDS is a part of a superfamily of lipid-binding proteins called lipocalins that also include Fabps. Together, these results suggest that lipid signaling pathways regulate normal sleep, and that lipid-binding proteins likely mediate both synthesis and transport of lipid-derived signaling cascades involved in sleep behavior.

Diurnal fluctuations in other lipid species have also been identified in the CNS. Time-of-day cycling has been observed broadly in mammalian brain for the major endocannabinoids anandamide and 2-arachidonoyl-glycerol (2-AG) [53]. Within the rat hippocampus, higher levels of anandamide have been observed during the dark period [53], [54], while higher levels of 2-AG were observed during the light period [53]. Further, fatty acid amide hydrolase (FAAH), the enzyme which catabolizes anandamide, has levels that also vary based on time-of-day within various regions of the brain [53], [55], suggesting that lipid-metabolism likely also varies over the light/dark cycle. Inhibition of FAAH has recently been shown to promote memory processing [56], implicating anandamide-metabolism in the regulation of memory formation. Oleoylethanolamide (OEA), a monounsaturated analogue of anandamide and an endogenous peroxisome proliferator activated receptor (PPAR)-α agonist, failed to show any diurnal fluctuations within the hippocampus [54]. However, post-training administration of OEA, or exogenous PPAR-α agonists, have recently been shown to enhance memory for inhibitory avoidance and Morris water maze tasks [57]. These data suggest that an increase in specific lipid-signaling pathways are able to enhance memory formation during the consolidation period, and together indicate that increased levels of anandamide or its derivatives are able to enhance memory formation.

Interestingly, Fabp7 has recently been shown to be an intracellular carrier of anandamide [58], raising the possibility that subcellular transport of certain lipid molecules, such as anandamide or OEA, via Fabps may activate PPAR-mediated transcription in the CNS. Indeed, various Fabps have already been shown to traffic specific ligands from the cytoplasm to nuclear receptors, such as PPARs, in order to induce transcriptional activation [59]-[62]. Since PPARs are involved in the circadian clockwork in various tissues and cell types [63]-[66], these data provide additional evidence for Fabp signaling in gating temporal and memory-enhancing processes.

Growing evidence suggests that sleep is critical for memory consolidation processes [67]. Previously, post-training temporal windows of sleep were found to be necessary for rodent memory [11], [68]. Improved cognitive performance has been found to correlate with sleep and “naps”, while deterioration in memory formation is observed when post-training sleep is disrupted [69]–[74]. Sleep has previously been shown to recover performance deficits observed in various cognitive tasks in humans [75], [76]. More recently sleep (and not merely the passage of time) has been suggested to enhance hippocampus-dependent memory in mice [77] and auditory discrimination performance in European starlings [78]. However, there are few examples of improved performance when post-training sleep is increased or otherwise enhanced. Two such studies involve pharmacological stimulation of REM activity and the “rescue” of sleep deprivation's disruptive effects on performance [79], [80]. While all of these studies point toward a role of sleep in memory consolidation, direct evidence for the affects of augmented sleep on memory formation has been lacking. Furthermore, evidence for genetic control of sleep with concomitant beneficial effects on cognitive ability has not been demonstrated. Here we show that Fabp enhances LTM concurrently with an increase in sleep during post-training consolidation periods in *Drosophila*. Since Fabp overexpression increases consolidated daytime sleep bouts, these data support a role for longer naps in the enhancement of memory. Further, these data suggest a late temporal “consolidation window” which occurs after the initial post-training ∼24 hour period usually associated with “synaptic consolidation”. It is conceivable that chronic Fabp-mediated increases in sleep could cause performance improvements during recall, providing an alternative to a pure consolidation effect. This question remains open for future study. Our experimental design also excludes memory improvement resulting from indirect effects on arousal, motivation, or stress during training and retrieval. Since our data is based on a simple associative behavioral task in an invertebrate model, the requirement for sleep in memory consolidation may be surprisingly broad. Finally, this work points toward a novel molecular link between sleep, memory formation, and fatty acid signaling, and may lead to new treatments relating lipid metabolism with sleep, learning, and cognitive disorders.

## Methods

### Transgenic Flies

The background strain *w(isoCJ1)* is an isogenic line previously described [29]. Murine Fabp7 open-reading frame (ORF) cDNA was generated by PCR using a forward primer containing the *Eco*RI restriction sequence and a Kozak sequence, and a reverse primer containing a *Bgl*II sequence. The resulting fragment was subcloned into the p(CaSpeR-hs) vector. An identical strategy was used for the *Drosophila* dFabp-B ORF. All plasmids were sequenced and injected into *w(isoCJ1)* embryos (BestGene, Inc.).

Fabp7 forward primer: 5′-ACCACCGAATTCCAACATGGTAGATGCTTTCTGCGCAACCTG -3′; Fabp7 reverse primer 5′- ACCACCAGATCTCTATGCCTTTTCATAACAGCGAACAGC-3′. dFabp forward primer: 5′- ACCACCGAATTCCAACATGTCTTTCGTTGGCAAGAAGTACAAG -3′; dFabp reverse primer 5′- ACCACCAGATCTTTAGACGGCCTTGTAGACGCGC -3′.

### Northern Blotting

Fly brain RNA was extracted using Trizol (Invitrogen) and Northern blot analysis done as described [27]. Briefly, brains were dissected and total RNA was isolated using TRIZOL (Invitrogen, Carlsbad, CA), according to the manufacturer's specifications, and stored at −80°C. Prior to loading, each sample was incubated with sample buffer (7.5%Formaldehyde, 43%Formamide, 12% 10XMOPS, 0.12%Ethidium Bromide) for 5 min at 56°C, and then cooled on ice for 5 min. Northern Blots consisted of 1 µg of total RNA per lane and were electrophoresed on a 1.2% agarose gel. RNA was transferred overnight onto a sheet of GeneScreen Plus nitrocellulose (NEN Life Science Products, Boston, MA). DNA templates generated by PCR were labeled with 32P using the Megaprime DNA labeling system (Amersham Biosciences, England). Antisense dFabp cDNA probes were PCR amplified from above products. *Drosophila* Gapdh forward primer: 5′ - TTTCTCAGCCATCACAGTCG - 3′; reverse primer: 5′ - CGACCTCCTCATCGGTGTAT -3′. Probe was mixed with hybrisol, and incubated overnight at 42°C. Blots were washed three times in 2× SSC (1×SSC  = 150 mM NaCl, 15 mM Na citrate, pH, 7.0), 1% SDS at room temperature, then two times for 30 min in 2×SSC, 1% SDS at 50°C, and finally one time for 30 min in 0.5×SSC, 1% SDS at 60°C. Following washes, blots were exposed to a phosphoscreen for 3 days and image analysis was performed using the Storm 860 and ImageQuant 5.2 software (Molecular Dynamics, Sunnyvale, CA).

### Immunocytochemistry

Fly brains were fixed and stained as described [81]. Antibodies against murine Fabp7 or dFabp-B were generated by purifying MAT-FLAG-tag (Sigma-Aldrich) fused ORFs expressed in *E.coli* using a metal affinity column, and injected into rabbits (Cocalico Biologicals, PA). Anti-Fabp7 or dFabp-B was diluted 1∶100, Topro-blue 1∶500. All images were captured with the Bio-Rad MRC-1024 Confocal System (Keck Imaging Center, UW-Madison).

### Western Blotting

Fly head extracts were lysed and Western blot analysis done as described [27]. Protein samples prepared were subjected to SDS-polyacrylamide gel electrophoresis by separation on a 12–15% gel, transferred to 0.2 µm Protran nitrocellulose membranes (PerkinElmer, Boston, MA), blocked in 5% dried milk powder in 50 mM Tris-HCl, pH 7.5, 15 mM NaCl, 0.5% Tween-20 (TBST), washed briefly in TBST and incubated in primary antibody in TBST overnight at 4°C. Fabp7 or dFabp antibodies were used at ∼1∶1500, laminin antibody (Hybridoma bank, U Iowa) ∼1∶1000. Blots were then washed 3 times in TBST, incubated for 1 hour in anti-rabbit horse radish peroxidase-conjugated secondary antibody (1∶7500 in TBST), washed 3 times in TBST and incubated in ECL plus Western blotting detection reagent for 5 minutes based on the manufacturer's instructions (Thermo Scientific, Rockford, IL). Visualization was performed by chemoluminescent exposure to X-Ray film (Eastman Kodak, Rochester, NY) and quantitation was done using ImageJ (NIH).

### Behavioral Testing

Flies were maintained on standard molasses-yeast-cornmeal food at 22°C, and entrained to a 12-h∶12-h light:dark cycle for 2-3 d before being assayed for sleep or olfactory avoidance conditioning. For analysis of sleep behavior, ∼3–4 day old female flies were assayed using the Drosophila Activity Monitoring System (Trikinetics), carried out as previously described [82]. Olfactory avoidance conditioning was done as described [29]. Briefly, the performance index (PI) was calculated by subtracting the number of flies making the incorrect choice from those making the correct one, dividing by the total number of flies, and multiplying by 100. To avoid odor-avoidance biases, we calculate the PI of each single n by taking an average performance of two groups of flies, one group trained with the CS+ being OCT, the other with the CS+ being MCH. Methylcyclohexanol was diluted 1∶100, Octanol was diluted 1∶40 in mineral oil. *w(isoCJ1)* and Fabp transgenics were combined for training and testing in the same environments at the same time for most sleep and memory experiments.

## Supporting Information

Figure S1 **Western blots of dFabp and Fabp7 protein in fly heads** (**A**) Western blots of fly head lysates showing the diurnal regulation of the dFABP band at 15kD, as compared to a loading control (laminin). 50-100 head equivalents of protein were loaded in each lane. (**B**) Western blots of *w(isoCJ1)* (w) background strain and Fabp7 overexpressing fly head lysates showing the presence and overexpression of Fabp7 using antibodies raised against Fabp7 at 20°C and 30°C. 50-100 head equivalents of protein per lane. ZT  =  zeitgeber time; RT  =  room temperature.
(EPS)

Figure S2 **Reduced sleep in multiple Fabp7 overexpressing fly strains.** Hourly sleep profile of the (A) *w(isoCJ1)* background strain, (B) a second Fabp7 (103-5) strain, and (C) a third Fabp7 (103-8) strain. Open bars  =  lights on, filled bars  =  lights off. Analysis of various sleep parameters: (D) Total sleep over 24 hours, (E) Total daytime sleep, (F) Average daytime sleep bout length, (G) Maximum daytime sleep bout length, (H) Daytime locomotor activity per waking minute. Open bars  =  *w(isoCJ1)*, light grey bars  =  103-5, dark grey bars  =  103-8. Results are mean ± s.e.m.; *n*  =  11-14, t, *P*≤0.0502; single asterisk, *P* < 0.05; double asterisk, *P* < 0.01; triple asterisk, *P* < 0.001, *t*-test.
(EPS)

Figure S3 **Reduced sleep in multiple dFabp overexpressing fly strains.** Hourly diurnal sleep profile of the (A) *w(isoCJ1)* background strain, (B) a second dFabp (101-2) strain, and (C) a third dFabp (101-6) strain. Open bars  =  lights on, filled bars  =  lights off. Analysis of various sleep parameters: (D) Total sleep over 24 hours, (E) Total daytime sleep, (F) Average daytime sleep bout length, (G) Maximum daytime sleep bout length, (H) Daytime locomotor activity per waking minute. Open bars  =  *w(isoCJ1),* light grey bars  =  101-2, dark grey bars  =  101-6. Results are mean ± s.e.m.; *n*  =  13 *w(isoCJ1), n*  =  22-24; single asterisk, *P* < 0.05; double asterisk, *P* < 0.01; triple asterisk, *P* < 0.001, *t*-test.
(EPS)

Figure S4 **Acquisition and retrieval in Fabp flies.** (A) Fabp7 (grey fill) and (B) dFabp (dark grey fill) flies exhibit normal learning after a single training trial (1X). Results are mean ± s.e.m.; *n*  =  8 groups. (C) An acute 30 minute 34°C heat shock (+) of Fabp7 flies 2 hours prior to testing inhibits LTM retrieval following 10X Spaced (10XSP) training of flies maintained at 20°C for 6 days, compared to non-heat shock (-) controls. Results are mean ± s.e.m.; *n*  =  8 groups. Asterisk *P*<0.05, *t*-test. (D) An acute 30 minute 34°C heat shock (+) of the *w(isoCJ1)* background strain flies 2 h prior to testing has no effect on LTM retrieval following 10X Spaced (10XSP) training of flies maintained at 20°C for 6 days, compared to non-heat shock (-) controls. Results are mean ± s.e.m.; *n*  =  8 groups. Grey bars, Fabp7, open bars, *w(isoCJ1)* background strain. d1  =  day 1, d6  =  day 6.
(EPS)
